# Early Presentation of Hyperinsulinism/Hyperammonemia Syndrome in Three Serbian Patients

**DOI:** 10.4274/jcrpe.2436

**Published:** 2016-06-06

**Authors:** Adrijan Sarajlija, Tatjana Milenkovic, Maja Djordjevic, Katarina Mitrovic, Sladjana Todorovic, Bozica Kecman, Khalid Hussain

**Affiliations:** 1 Mother and Child Health Care Institute of Serbia “Dr Vukan Cupic”, Department of Metabolism and Clinical Genetics, Belgrade, Serbia; 2 University of Belgrade Faculty of Medicine, Belgrade, Serbia; 3 Mother and Child Health Care Institute of Serbia “Dr Vukan Cupic”, Department of Endocrinology, Belgrade, Serbia; 4 Great Ormond Street Hospital for Children NHS Trust, Department of Pediatric Endocrinology, London, United Kingdom; 5 University College London, Institute of Child Health, London, United Kingdom

**Keywords:** Hyperinsulinism/hyperammonemia syndrome, genotype, phenotype

## Abstract

Hyperinsulinism/hyperammonemia (HI/HA) syndrome is considered as the second most common type of hereditary HI. Correlation of genotype and phenotype in HI/HA syndrome has been described in several studies. We present three Serbian patients with HI/HA syndrome with emphasis on a possible correlation between genotype and clinical manifestations. Patient 1 was heterozygous for a de novo mutation p.S445L in the GLUD1 gene, while patients 2 and 3 (son and mother) both carry the p.R221C mutation. Early onset of hypoglycaemia with generalized seizures was recorded in infancy in all three patients. The two male patients had mild developmental delay, while the female patient presented with epilepsy. Analysis of Serbian patients with HI/HA syndrome confirms the association of p.S445L and p.R221C mutations with hypoglycaemic seizures noted within the first three months of life and with subsequent risk for cognitive impairment and/or epilepsy.

**WHAT IS ALREADY KNOWN ON THIS TOPIC?**

Hyperinsulinism/hyperammonemia (HI/HA) syndrome is considered as the second most common type of hereditary HI. Dominantly expressed, activating mutations in GLUD1 gene encoding glutamate-dehydrogenase are responsible for occurrence of HI/HA syndrome. Mutations in exons 6 and 7 tend to be associated with increased risk of epilepsy, but genotype-phenotype correlation was also noted for other characteristics of HI/HA syndrome patients (e.g. age of onset, birth weight).

**WHAT THIS STUDY ADDS?**

Patients carrying p.R221C mutation may present with hypoglycaemic seizures earlier than previously reported with increased risk for cognitive impairment and/or epilepsy at later age. The carriers of p.S445P mutation could be prone to early hypoglycaemic seizures and mild developmental delay.

## INTRODUCTION

Hyperinsulinism/hyperammonemia (HI/HA) syndrome is considered as the second most common type of hereditary HI (1). Dominantly expressed, activating mutations in GLUD1 gene encoding glutamate-dehydrogenase (GDH) are responsible for occurrence of HI/HA syndrome (2). It was estimated that nearly 80% of disease-causing mutations arise de novo in GLUD1 gene (3). First clinical manifestations are usually hypoglycaemic seizures with median age of onset ranging from 4 to 11 months according to different studies (1). During the course of the disease, recurrent symptomatic hypoglycaemia, both during fasting and after protein-rich meals, remains the main clinical feature. Plasma ammonia levels are commonly elevated 2-5 fold the upper limit, but HA is mostly considered as benign due to the specific pathophysiology of the HI/HA syndrome (1). On the other hand, a high prevalence of epilepsy and delayed mental development are reported in these patients (4,5,6,7). The risk for epilepsy is not in correlation with HA but is speculated to be the consequence of altered GDH activity in the brain (8). Mutations in exons 6 and 7 tend to be associated with increased risk of epilepsy (4,5,9). Genotype-phenotype correlation was also noted for other characteristics of HI/HA syndrome patients such as age of onset and birth weight (4,5). Diazoxide treatment of hypoglycaemia in patients with HI/HA is usually successful (3).

We present three Serbian patients with HI/HA syndrome with emphasis on possible correlation between genotype and clinical manifestations.

## CASE REPORTS

Patient 1 is the second male child of healthy non-consanguineous parents. He was born at term with a birth weight of 3850 g, birth length of 51 cm, and an Apgar score of 10 at one minute. At the age of nine weeks, the infant had generalized tonic-clonic seizures. Upon admission, the blood glucose level was 2.0 mmol/L with a simultaneous serum insulin level of 32.4 mIU/L. The plasma ammonia level was of 277 μmol/L (reference range 11-50 μmol/L). During the following days, the infant had recurrent hypoglycaemia and persistently high ammonia levels ranging from 219 to 315.5 μmol/L. There were no abnormalities in plasma amino acid levels nor in urinary organic acid concentrations. Mild hypotonia was observed at 9 weeks of age. At this time, the infant’s weight had increased to 6.9 kg (92nd centile) and length to 62 cm (75th centile). Electroencephalography (EEG) pattern was normal. Family history for hypoglycaemia was negative.

Genetic analysis was performed in the Molecular Genetics Laboratory of Exeter and Plymouth Universities (UK), and the patient was found to be heterozygous for de novo missense mutation p.S445L (c.1334C'T) in exon 11 of GLUD1 gene. Initial treatment included diazoxide (5 mg/kg/day) and hydrochlorothiazide (1 mg/kg/day) with advice for avoiding protein-rich meals. During the following four months, hypoglycaemic episodes were verified once in two months, usually after increased protein intake. Diazoxide dose was increased to 7 mg/kg/day, while the patient was weaned off hydrochlorothiazide therapy. At 5 months of age, the boy was overweight with 8.6 kg (93rd centile), and at 8 months, he weighed 9.85 kg (88th centile). However, at age 30 months, his body mass index was within normal limits (17.49 kg/m2, 82nd centile). The boy walked independently at 14 months of age. A borderline delay in mental development was verified at 30 months with a developmental quotient (DQ) of 85 according to Brunet-Lézine scale. After introduction of diazoxide, the boy remained seizure-free.

Patient 2 was admitted to a local hospital due to generalized seizures at the age of 10 weeks. Hypoglycaemia (2.4 mmol/L) was verified, but no further investigations were done at the time. This male infant was the first child of non-consanguineous parents, born at term, with a birth weight of 3050 g, a birth length of 49 cm, and an Apgar score 9 at one minute. At the age of six months, seizures reoccurred with hypoglycaemia (1.4 mmol/L), and the patient was referred to our Institute. On admission, the infant had normal physical findings with a weight of 8200 g (62nd centile) and length of 68 cm (50th centile). During the first day of hospitalization, the lowest value of glycaemia was 2.8 mmol/L with a concomitant insulinemia of 19.82 μmol/L and HA of 159.2 μmol/L. EEG pattern was described as normal. Treatment was started with diazoxide (5 mg/kg/day) and hydrochlorothiazide (2 mg/kg/day) with advice for avoiding protein-rich meals. Since the patient missed regular check-ups, the next examination was performed at 20 months of age and speech delay with mild hyperactivity was noted. The parents acknowledged several hypoglycaemic crises (non-seizure) early after treatment introduction. At the age of 3.5 years, physical status was normal while neuropsychological assessment confirmed borderline mental delay (DQ 90 at Brunet-Lézine scale) associated with signs of attention-deficit/hyperactivity disorder.

Patient 3 (the mother of patient 2) was discovered by the family history which revealed that she had presented to a hospital at age 12 weeks with generalized tonic-clonic seizures. The blood glucose level was not measured during the initial investigation in this local hospital. Her perinatal history was uneventful. Her birth weight was 2800 g. At the age of six months, this patient was referred to our Institute due to recurrent seizures and at admission, severe hypoglycaemia of 0.6 mmol/L was verified. Further testing revealed HI (an insulin level of 59.8 pmol/L associated with hypoglycaemia of 1.0 mmol/L). Diazoxide was initiated (10 mg/kg/day) and very good control of glycaemia was achieved in the following years. From the second year of life on, the patient’s seizures were mostly episodes of absence with eyelid myoclonia and occasional generalized tonic-clonic seizures. The epileptic state worsened at the age of 15 years requiring administration of two antiepileptic drugs but without achieving complete seizure control. Currently, she experiences several generalized tonic-clonic seizures per year (usually after protein-rich meals) and continues to have eyelid myoclonic seizures at least once weekly. She became increasingly non-compliant to diazoxide treatment during her late teenage period and stopped taking this medication at 18 years of age without consulting her physician. Ammonia was tested for the first time in her adult age, and values ranged from 100 to 120 μmol/L. The patient is a housewife who graduated from a regular high school, however, her intellectual level was not clinically estimated at adult age.

Genetic analysis performed in the Molecular Genetics Laboratory of Exeter and Plymouth Universities (UK) showed that the boy and his mother (patients 2 and 3) are heterozygous for mutation p.R221C (c.833C>T) in exon 6 of GLUD1 gene.

## DISCUSSION

We have identified three patients with HI/HA syndrome from two Serbian families. These are the first cases of HI/HA syndrome reported from Serbia. Patient 1 manifested with hypoglycaemic generalized seizures at the 10th week of life, a finding which is in accordance with reports that p.S445P is associated with early onset of HI/HA syndrome. In other words, generalized tonic-clonic seizures occurred between the 1st week and 5th month of life in all patients with p.S445P reported by Kapoor et al (4), Bahi-Buisson et al (5), Raizen et al (6), and Diao et al (7).

In one study, it was observed that p.S445P mutation was associated with epilepsy in one third of patients and also with borderline to mild development delay in all patients. Thus, our patient showed a neurologic pattern showing similarities to previous reports (5). However, the follow-up period for patient 1 is still relatively short since he is only 3 11/12 years old at the time of this report.

Patient 1 harboring a p.S445P mutation exhibited higher peak plasma ammonia concentration than our patients with p.R221C mutation. This observation is also in accordance with previous reports (4). As expected, the level of the HA did not correlate with neurological outcome in our HI/HA patients.

Early onset of generalized tonic-clonic seizures associated with frank hypoglycaemia was noted in patient 2. In patient 3, carrying the same p.R221C mutation, generalized seizures also occurred as early as three months of age. Carriers of this mutation have been previously reported to have neonatal disease onset (4). However, patients with p.R221C mutation usually have somewhat later disease onset (7-23 months) or stay asymptomatic until adulthood as suggested by Bahi-Buisson et al (5).

Our patient 2 has a borderline developmental delay noted during the second year of life, while epilepsy did not occur during the follow-up period of 3 years and 10 months. His mother (patient 3), who had very similar initial presentation, developed absence epilepsy with eyelid myoclonia during her second year of life. Bahi-Buisson et al (5) reported a strong association of p.R221C mutation with absence and eyelid myoclonia epilepsy starting between 2 and 6 years of age, refractory to treatment in 40% of cases. A similar course of epilepsy was also noted in our patient 3. The same study reported hypoglycaemic seizures starting at 7-23 months as a first manifestation of HI/HA syndrome caused by p.R221C mutation, with borderline to moderate learning disability in the majority of these patients. An earlier study described one family with five members affected by HI/HA syndrome due to p.R221C mutation (10). Similar to other studies, in this family, first symptoms are reported to occur between 6 and 15 months of age, and all patients who survived until adulthood had mental retardation, while 50% of them had epilepsy. Our findings also suggest a risk for mild cognitive impairment, attention deficit, hyperactivity, and refractory epilepsy for carriers of p.R221C mutation. However, our patients had an earlier onset than the majority of previously reported cases.

Apart from studies of HI/HA syndrome, investigations of cohorts with congenital HI (CHI) showed a substantial prevalence of mental retardation (26-44%) and epilepsy (17.7-25%) in this population (11,12). Interestingly, these researchers reported conflicting results regarding age of onset as a risk factor for mental retardation in CHI patients. A case series study of HI/HA syndrome patients suggested that neonatal onset of the disease could be a risk factor for occurrence of infantile spasms (13). The largest study on neurological outcome in patients with HI/HA syndrome showed that onset within the first 3 months of life was associated with borderline to mild developmental delay in all patients and with epilepsy in more than 70% (5).

Incompliance to diazoxide treatment has led to worsening of epilepsy in our patient 3. One recent report pointed out the success of diazoxide treatment in inducing remission of epileptic seizures in both pediatric and adult patient from the same family (9). All three of our patients had a good response to diazoxide treatment, a finding which is in agreement with the findings of others (4,5,6,7). We did not perform a protein loading test, so the recommendation to avoid protein-rich meals was given on the basis of previous studies of HI/HA syndrome (1,14). Japanese authors reported a patient with mutation in exon 7 of GLUD1 gene who presented with absence epilepsy and eyelid myoclonia refractory to treatment and whose seizures intensified after protein-rich meals (15). Patient 3 from our series also experienced seizures after higher intake of proteins, in absence of hypoglycaemia. Avoidance of protein-rich meals appears to be necessary through adulthood, especially after the cessation of diazoxide therapy.

Proposed pathogenic mechanisms for epilepsy and developmental delay in HI/HA syndrome include effects of chronic HA, hypoglycaemic brain injury, and decreased brain tissue concentration of glutamate and gamma-aminobutyric (GABA) acid due to GDH hyperactivity (4,16). So far, there is no evidence for correlation between serum concentration of ammonia and the risk for epilepsy and/or intellectual impairment in patients with HI/HA syndrome (5). A typical pattern of ammonia toxicity to the brain is not present in HI/HA syndrome since hyperactivity of GDH prevents brain oedema by decreasing intracellular concentration of glutamine. Similarly, correlation of frequency and severity of hypoglycaemia to neurological outcome has not been proven in previous studies of this disorder (6,16). On the other hand, hyperactive GDH causes depletion of glutamate in brain cells leading to disturbed synthesis of GABA which acts as the key inhibitory neurotransmitter (16). It seems that pathogenetic mechanisms involved in occurrence of epilepsy and developmental delay in HI/HA syndrome are somewhat different than in patients with other types of CHI. Higher prevalence and earlier occurrence of neurological complications in patients with HI/HA syndrome when compared to other causes of CHI syndrome prompts a need for further elucidation of its pathophysiology in future studies.

In summary, we believe that an analysis of Serbian patients with HI/HA syndrome could contribute to a better understanding of genotype-phenotype correlations in this rare disease. Namely, we have shown that patients carrying p.R221C mutation may present with hypoglycaemic seizures earlier than previously reported with increased risk for epilepsy and/or cognitive impairment. Data from previous studies as well as from our case series suggest that neurologic outcome in patients with HI/HA syndrome depends on a multitude of factors, including genotype, age of onset, and compliance to dietary and drug treatment.

## Ethics

Informed Consent: It was taken.

Peer-review: External peer-reviewed.
